# RETRACTION: Comparison of Endovenous Laser Treatment and High Ligation in Treatment of Limb Varicosity: A Meta‐Analysis

**DOI:** 10.1111/iwj.70635

**Published:** 2025-04-23

## Abstract

**RETRACTION:** CaoG. , GuH.C. , WangJ.T. , HuangQ. , and CaoJ.C. , “Comparison of Endovenous Laser Treatment and High Ligation in Treatment of Limb Varicosity: A Meta‐Analysis,” International Wound Journal 16, no. 3 (2019): 696–702, 10.1111/iwj.13083.30767406 PMC7949302

The above article, published online on 15 February 2019, in Wiley Online Library (http://onlinelibrary.wiley.com/), has been retracted by agreement between the journal Editor in Chief, Professor Keith Harding; and John Wiley & Sons Ltd. Following an investigation by the publisher, all parties have concluded that this article was accepted solely on the basis of a compromised peer review process. The editors have therefore decided to retract the article. The authors did not respond to our notice regarding the retraction.

**RETRACTION:** G. Cao , H.C. Gu , J.T. Wang , Q. Huang , and J.C. Cao , “Comparison of Endovenous Laser Treatment and High Ligation in Treatment of Limb Varicosity: A Meta‐Analysis,” International Wound Journal 16, no. 3 (2019): 696–702, 10.1111/iwj.13083.30767406 PMC7949302

The above article, published online on 15 February 2019, in Wiley Online Library (http://onlinelibrary.wiley.com/), has been retracted by agreement between the journal Editor in Chief, Professor Keith Harding; and John Wiley & Sons Ltd. Following an investigation by the publisher, all parties have concluded that this article was accepted solely on the basis of a compromised peer review process. The editors have therefore decided to retract the article. The authors did not respond to our notice regarding the retraction.

